# Social and geographical factors influencing the delay in treatment for colorectal cancer

**DOI:** 10.1038/sj.bjc.6602170

**Published:** 2004-09-21

**Authors:** O Dejardin, C Herbert, M Velten, A Buemi, F Ménégoz, N Maarouf, G Launoy

**Affiliations:** 1 ‘Cancers & populations’ ERI3 INSERM Faculté de médecine, 14032 Caen Cedex, France; 2 FRANCIM (French network of cancer registries), France

**Sir**,

In a recent issue, Robertson *et al* (2004) reported on the time from presentation to treatment of colorectal and breast cancers in Scottish urban and rural areas.

Using as their principal outcome, the time from first presentation with suspicious symptoms or signs to treatment, there was no evidence that people living in urban areas received treatment more quickly. Furthermore, delay did not vary according to hospital type or distance from residence to the nearest cancer centre. However, age and number of female GPs (practice level) were significantly associated with a reduction of delay. In a previous issue, Campbell *et al* (2002) found significant difference in the delay between people living far from a cancer centre (more than 58 km) and those living near a cancer centre (less than 5 km).

We recently conducted a similar study focused on people with colorectal cancer diagnosed in 1995 in five French departments covered by a cancer registry (Calvados, Isère, Manche, Bas-Rhin and Haut-Rhin). We used as principal outcome the time from first specialist presentation to treatment (surgery, chemotherapy or radiotherapy). The main independent variables studied were: road distance to specialized cancer units (University hospital and cancer care centre), occupation, marital status, gender, place of residence (urban *vs* rural), cancer stage, hospital type, emergency admission and first specialist referral. Unlike Robertson *et al* (2004), we preferred to used the Cox hazard model in order to include in the analysis patients without treatment (*N*=40).

The mean delay was 27.9 days. Since we found no influence of place of residence (urban *vs* rural), distance to specialised cancer centre and occupation on delay, these variables were not included in the final model. Emergency admission and surgeon as first specialist referral were associated with a shorter delay (Table 1

**Table 1:** Time between first specialist referral and treatment (Cox hazard model final regression)

***N*=903**	***N***	Odds ratio*	**95% confidence interval**		**Standard error**	*P*-values**
*Age*						
<65	259					
65–74	317	1.00	0.84	1.19	0.09	NS
75–84	229	0.99	0.82	1.19	0.10	NS
>84	97	0.92	0.71	1.17	0.13	NS
Unknown	1					

*Sex*						
Male	491					
Female	412	1.07	0.93	1.22	0.07	NS

*Cancer stage*						
Dukes A, B or C	655					
Métastasis or not operable	215	0.66	0.56	0.78	0.09	^***^
Unknown	33	1.04	0.73	1.48	0.18	NS

*Department of residence*						
Calvados	226					
Isere	122	1.09	0.87	1.37	0.12	NS
Manche	186	0.83	0.63	1.11	0.14	NS
Bas-Rhin	192	1.36	1.11	1.66	0.10	^***^
Haut-Rhin	177	0.82	0.62	1.09	0.14	NS

*Emergency admission*						
No	756					
Yes	130	2.43	1.92	3.08	0.12	^***^
Unknown	17	1.21	0.73	2.01	0.26	NS

*Type of first referral*						
Gastro-enterologist	533					
Surgeon	138	1.62	1.27	2.05	0.12	^***^
Other specialist	113	0.91	0.73	1.14	0.11	NS
Unknown	119	1.09	0.88	1.34	0.11	NS

* Odds ratio higher than unity means a shorter delay before treament.

** Significant levels are: ^*^<0.10; ^**^<0.05; ^***^<0.01; NS=not significant.

). Patients living in Bas-Rhin department had a shorter delay than patients living in Calvados department. Advanced stage of cancer (metastases and inoperable) was significantly associated with longer delay, probably due to a more complex management.

Health care system and health services are notably different in France and Scotland. Nevertheless, except for minor details, our study exhibits results similar to those shown by Robertson and Campbell: the delay from first presentation to treatment is slightly shorter in France than in Scotland, but more importantly, in both countries, there is no relationship between social or geographical variables and the delay before treatment.

Social inequalities in cancer survival are well established in different countries (Auvinen and Karjalainen, 1997). The Scottish and French data suggest that the delay from presentation to treatment does not contribute to the social differences in survival. Further studies are thus needed to confirm the possible contribution of social differences in access to specialised care centre.
